# Image quality evaluation in a modern PET system: impact of new reconstructions methods and a radiomics approach

**DOI:** 10.1038/s41598-019-46937-8

**Published:** 2019-07-23

**Authors:** Gabriel Reynés-Llompart, Aida Sabaté-Llobera, Elena Llinares-Tello, Josep M. Martí-Climent, Cristina Gámez-Cenzano

**Affiliations:** 1 Medical Physics Department, Institut Català d’Oncologia, L’Hospitalet de Llobregat, Barcelona, Spain; 2 PET Unit. Nuclear Medicine Dept, IDI. Hospital U. de Bellvitge-IDIBELL, L’Hospitalet de Llobregat, Barcelona, Spain; 3 Medical Physics Department, Clínica Universidad de Navarra, Pamplona, Spain

**Keywords:** Cancer imaging, Molecular medicine

## Abstract

The present work investigates the influence of different biological and physical parameters on image quality (IQ) perception of the abdominal area in a modern PET scanner, using new reconstruction algorithms and testing the utility of a radiomics approach. Scans of 112 patients were retrospectively included. Images were reconstructed using both OSEM + PSF and BSRM methods, and IQ of the abdominal region was subjectively evaluated. First, 22 IQ related parameters were obtained (including count rate and biological or mixed parameters) and compared to the subjective IQ scores by means of correlations and logistic regression. Second, an additional set of radiomics features was extracted, and a model was constructed by means of an elastic-net regression. For the OSEM + PSF and especially for the BSRM reconstructions, IQ parameters presented only at best moderated correlations with the subjective IQ. None of the studied parameters presented a good predictive power for IQ, while a simple radiomics model increased the performance of the IQ prediction. These results suggest the necessity of changing the standard parameters to evaluate IQ, particularly when a BSRM algorithm is involved. Furthermore, it seems that a simple radiomics model can outperform the use of any single parameter to assess IQ.

## Introduction

Positron emission tomography (PET) with ^18^F-2-fluoro-2-deoxy-D-glucose (FDG) has become a routine image procedure for the management of oncological patients. Compared to other imaging modalities, PET exams are limited by their low spatial resolution and signal-to-noise ratio (SNR)^1^. However, there is still an interest in decreasing as much as possible the administered activity dosage, both for patient safety and economic concerns, though images must maintain a certain level of diagnostic accuracy, not only in clinical research and trials, but also for medical diagnostic purposes.

The assessment of image quality (IQ) in PET is a challenging task affected by biological and physical factors^2^. It can be studied using phantoms or human examinations by means of different quantitative metrics. Some of the measurements could be considered as standard IQ parameters, such as the variance, SNR or contrast-to-noise ratio (CNR) of a target region (commonly a lesion or part of the healthy liver when human beings are involved)^3–5^. These parameters are an objective measurement that allows automation, though there can lack a connection between them and how IQ is perceived by the physician in some defined tasks (e.g. organ definition).

Beyond the reconstructed image, there is another set of IQ parameters derived from the count statics of a PET study. The measurement of the patient noise equivalent count rate (NECR) is a promising idea in order to predict IQ, providing a measure of the image count statistics corrected by the degrading scatter and random events. Several studies have reported a relationship between NECR and other IQ parameters^6–10^, including IQ perception^11^. However, in everyday practice, clinical NECR is not used to optimize clinical protocols or to establish a minimum level of IQ for clinical trials, probably due to the high uncertainties involved in its IQ prediction.

Another form of IQ assessment is via model-based tasks using automated models. Nevertheless, qualitative tasks are difficult to automate, as they involve a subjective human assessment, and models are usually limited to lesion detectability and conspicuity^12,13^.

In recent years, one of the main gains in IQ comes from advances in reconstruction methods. The inclusion of point-spread function (PSF) modeling in the iterative methods supposed an improvement in terms of diagnostic performance, though the relation between count statistics and IQ increased in complexity^7^. Additionally, penalized reconstruction methods were recently introduced into clinical practice. In contrast to ordered subset expectation maximization (OSEM), block sequential regularized expectation maximization (BSREM) methods can run until full convergence while controlling noise levels^14–16^. The penalization acts as a selective filtering and the level of noise or IQ could be rather different than OSEM with PSF algorithms. The reliability of predicting IQ using the aforementioned assessment methods, such as the SNR or NECR, has never been tested on these new reconstruction algorithms.

Despite all factors that could affect IQ in PET studies, dosage optimization of the administered activity is usually calculated only in terms of patient weight^17^. Once the acquisition starts, the only relevant parameters that have a direct impact on image quantification are the acquisition time and image reconstruction settings, the latter being delicate to modify^18^.

A fast and automated model to predict IQ could optimize the acquisition and reconstruction parameters in real time, or serve as a basic metric to compare acquisitions in multicentric studies. Thus, this task could benefit the emerging field of radiomics, which intends to extract and process a large number of quantitative features from radiological images^19^. Automated IQ evaluation using these methodologies has been developed for brain and liver magnetic resonance imaging (MRI)^20,21^; however, there is still a lack of research on this topic in nuclear medicine imaging.

The present study has two main aims, and hence the manuscript is divide in two parts: the first one is to investigate the influence of different biological and physical parameters on IQ perception of the abdominal area using new algorithms (OSEM + PSF and BSREM) and a modern PET scanner (Discovery IQ); the second one aims to test the utility of a radiomics approach in the first task. The study is focused on the abdominal region as the presence of different anatomic structures, sometimes with low SNR and definition, makes it a complex area to evaluate in PET studies.

## Material and Methods

We obtained approval from the Bellvitge University Hospital Institutional Review Board. All work was done in accordance with institutional guidelines and regulations. This manuscript has been revised for its publication by the Clinical Research Ethics Committee of Bellvitge University Hospital. Written informed consent was waived by this Committee, as it was a retrospective analysis of our usual everyday work. The data of the patients were anonymized for the purposes of this analysis. The confidential information of the patients was protected according national normative.

### Patient selection, image acquisition and reconstruction

A total of 112 patients were retrospectively included. Patients were selected sequentially from torso oncological FDG PET/CT studies; a detailed description of its referral reason can be seen on Table 1. Exclusion criteria were: a blood glucose level higher than 200 mg/dl, an uptake time outside the range of 60–100 min after FDG injection, and any abnormal condition such as artifacts or lesions which averted a correct evaluation of the abdominal region.

**Table 1:** Clinical characteristics of the studied population and referral reason for the PET/CT scan.

Characteristics	
Age (years), median (range)	66 (19–86)
Sex, no. (%)	
Male	58 (52%)
Female	54 (48%)
Referral reason, no. (%)	
Lung	25 (20%)
Gynecologic	25 (20%)
Colorectal	21 (19%)
Lymphoma	12 (11%)
Skin Cancer	9 (8%)
Head and Neck	5 (4%)
Unknown Primary	4 (3%)
Hepatobiliary	4 (3%)
Urologic	4 (3%)
Breast	3 (3%)

PET/CT acquisitions were performed according to the EANM 2.0 guidelines^17^. Patients were injected with 2.7 MBq/kg and scanned at 2 min/bed position. All data were acquired on a Discovery IQ 5-ring PET/CT^22^ (GE Healthcare, Waukesha). Mean injected activity was 194 MBq (range 97–374 MBq) and mean uptake time was 68 min (range 60–98 min). Overlap between beds was 19%.

Two different reconstructions were used: an OSEM iterative reconstruction with modeling PSF (OSEM + PSF), commercial name VUE Point HD-Sharp (VPHD-S, GE Healthcare, Waukesha), using 12 subsets, 4 iterations and a 4.8 Gaussian post-filtering; and a BSRM penalized algorithm with PSF correction, Q.Clear (GE Healthcare, Waukesha), using a β value of 350, which is a validated penalization value for torso oncological examinations^16^. Both algorithms used an image matrix of 256 × 256 and CT based attenuation correction, as well as dead time, random, and scatter events corrections.

### Subjective image quality evaluation

**Figure 1:**
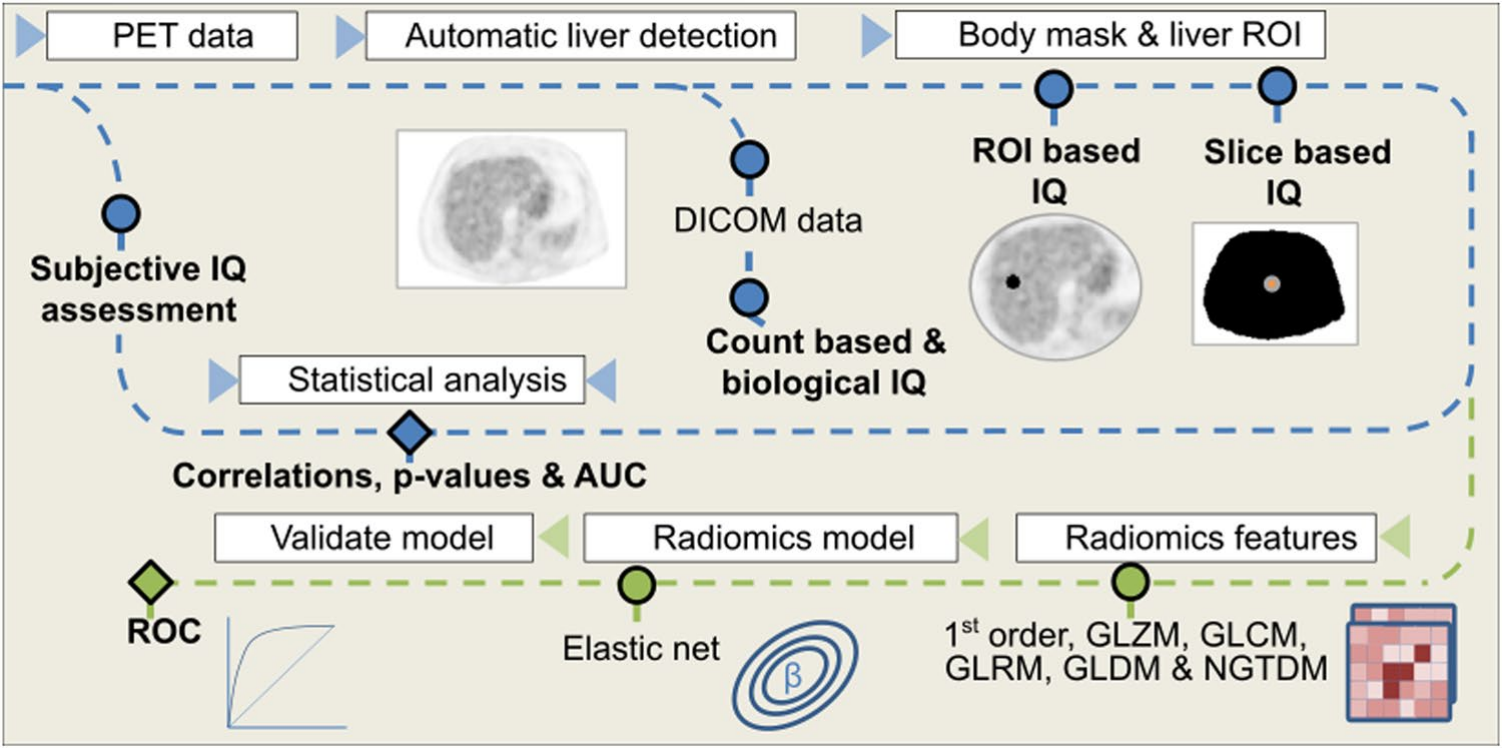
All image quality features were extracted and processed using an automatic pipeline. Blue line describes the first phase of the methodology: image is converted to SUV units and an automatic algorithm detects the slice including more liver parenchyma. Then, all DICOM data are extracted from the bed corresponding to this slice and a region of interest is placed on the liver to extract ROI-based image quality metrics. From a body mask, all slice-based image quality parameters are extracted. The green line describes the second phase: all common radiomics features are also extracted from the selected slices, as well as from its surrounding volume. Next, an elastic-net model is fitted selecting the relevant features. Results are compared in both lines with the subjective assessment.

Images were transferred to a dedicated review platform (AW Server 2.0) (GE Healthcare, Waukesha). IQ perception was evaluated by two different expert nuclear medicine physicians; both rankers had more than two years of clinical experience using the BSRM and OSEM + PSF reconstructions in the PET/CT system. Figure 1 summarizes the workflow for the extraction and processing of all data. Physicians were asked to evaluate the IQ of the axial slices of the abdominal area (IQ_ABD_) considering the conspicuity of the structures and the apparent noise. The score was ranked from 1 to 5 (1 non-diagnostic IQ, 2 poor IQ for diagnosis, 3 acceptable IQ but could lead to some undetermined judgment, 4 good IQ, and 5 excellent IQ). Moreover, all images were visualized in a randomized order mixing both reconstructions. The IQ_ABD_ was also grouped between low diagnostic quality (LQ) (1–3.5 score) and high diagnostic quality (HQ) (>3.5 score) to obtain a binary problem.

### Image quality features extraction

All data were processed with an in-house software programmed using Python 3.7 that automatically detected the bed containing more liver parenchyma, which was defined as the abdominal bed. As the Discovery IQ has an axial field-of-view (FOV) of 26 cm, it is a reasonable assumption that a single bed will include a major part of the liver. Table 2 presents all studied variables. All parameters were obtained from the data available in the DICOM header and from the image.

**Table 2:** Pre- and post-image reconstruction considered IQ parameters.

Biological	Count related	Mixed
**Pre-image reconstruction**		
Age, glucose level, body weight, body height, BMI, LBM	Activity at scan time, true count rate, random count rate, scatter rate, NECR, PNECR	Uptake time, R_DW_, R_DBMI_, R_DLBM_
**Post-image reconstruction**		
**Noise related**	**Radiomics**	**Others**
Variance_ROI_, SNR_ROI_, Variance_Slice_, SNR_Slice_ and CNR	1^st^ order, GLCM, GLZM, GLRM, GLDM, NGTDM	Patient position misplacement (center shift)

First, the image was loaded and converted to SUV units (Fig. 1). The slice containing the most liver parenchyma was automatically detected by using some heuristics on the suspected position range and the expected SUV values from healthy liver. More details of the used method can be found on supplemental data. Once the slice was defined, all count data and patient (biological) related data were extracted from the corresponding slice DICOM data. Additionally, an automatic region-of-interest (ROI) was placed in the healthy liver to account for SUV variance and SNR.

Next, a segmentation of the patient body in the liver slice was performed using a thresholding method followed by a morphological processing, which provides a mask used to perform all non-ROI based measurements. The same mask was used to fit the minimum circle around the abdominal surface and find the patient position misplacement (center shift).

Biological parameters that could potentially affect IQ included the age of the patient, glucose level at the injection time, patient height and weight, and uptake time. Body mass index (BMI) and lean body mass (LBM) were also computed, the second one defined as recommended by EANM 2.0 guidelines^17^, according to Janmahasatian equation^23^, which depends on patients’ sex.

NECR was computed directly over the total prompts, the random events, and the scatter factor extracted from manufacturer’s data inside the relevant DICOM tags, using the formulation provided by the NEMA standards^24^, defined as

1 $$NECR=\frac{{T}^{2}}{T+S+R}.$$

No additional corrections were used over these data, as could be the extraction of all count outside the body^6,9^. A metric closely related to the NECR was also used, called pseudo-NECR (PNECR) which was directly obtained from the sinogram and proportional to the NECR^6^, and defined as

2 $$PNECR=\frac{{(T+S)}^{2}}{T+S+R/2}.\,$$

An additional set of mixed parameters that combine count and biological parameters was considered. The ratios between activity at the acquisition start time and patient weight, BMI and LBM were also computed, defined as R_DW_^11^, R_DBMI_^11^ and R_DLBM_, respectively.

The mean value and variance were measured in the healthy liver ROI and in the body mask. SNR was measured dividing the mean value and standard deviation. CNR was measured using the ROI mean value and the mean and standard deviation of the mask. Lastly, from the body mask, the minimum surrounding circumference was extracted to compute the patient positioning shift (center shift, to abbreviate).

Radiomics features were extracted using the pyradiomics package^25^ from the same body mask described in the previous paragraphs. Moreover, the mask was extended to the two consecutive slices in both cranial and caudal directions to obtain a 3-dimensional mask, which will be referred as zone features. The extracted features are defined in compliance with feature definitions as described by the Imaging Biomarker Standardization Initiative (IBSI)^26^. A fixed bin number of 64 was used for feature extraction, employed in previous studies showing good reproducibility^27^.

### Investigations using standard parameters

All parameters listed in Table 2, excluding the radiomics features, were correlated to the IQ_ABD_ for both algorithms. Furthermore, this metrics were also compared using a two tailed Wilcoxon signed-rank test. Next, we studied the predictive power of all relevant parameters. All data was randomly split in a training (n = 73) and a test (n = 39) set. For each statistically significant value (p-value < 0.05) a logistic regression was fitted on the train data. Predicted IQ_ABD_ was computed for the test and train datasets, and the area under the curve (AUC) was obtained from the Receiver Operating Characteristic (ROC) curve.

### Building a Predictive Radiomics Model

To build a radiomics model to assess the IQ perception, the same train and test datasets were used. All Table 2 parameters were initially included in the radiomics model. This model consists of all IQ related parameters, as well as the texture parameters from the 1st order statistics (19 features), Gray Level Co-occurrence Matrix (GLCM, 24 features), Gray Level Size Zone Matrix (GLZM, 16 features), Gray Level Run Length Matrix (GLRM, 16 features), Gray Level Dependence Matrix (GLDM, 14 features), and Neighbouring Gray Tone Difference Matrix (NGTDM, 5 features). Non-normal features were log transformed. All features were standardized, by subtracting to each value the variable mean and dividing by the standard deviation. As the number of patients is limited, we used an algorithm to perform a feature reduction. First, Spearman’s rank correlation coefficients were calculated to examine the internal correlation between individual features. Redundant features with linear correlation coefficients >0.95 were removed. Then, an elastic-net feature selection approach and model building was adopted, which is a combination of the least absolute shrinkage selection operator (LASSO) and the Ridge Regression, and is suitable for the regression of high-dimensional data^28^. The LASSO shrinks all regression coefficients towards zero to set the coefficients of non-contributing features to exactly zero. To find an optimal penalization terms, a ten-fold cross validation with minimum criteria was used in the elastic-net parameter tuning. The retained features with non-zero coefficients were used for regression model fitting and combined into a radiomics signature. Different models were computed for the OSEM and BSRM algorithms.

The performance of the model was reported using the ROC methodology and AUC values in the training and test sets. The regression and its validation were performed using the R software version 3.4.4 (the R foundation) through the caret and glmnet packages.

## Results

**Figure 2:**
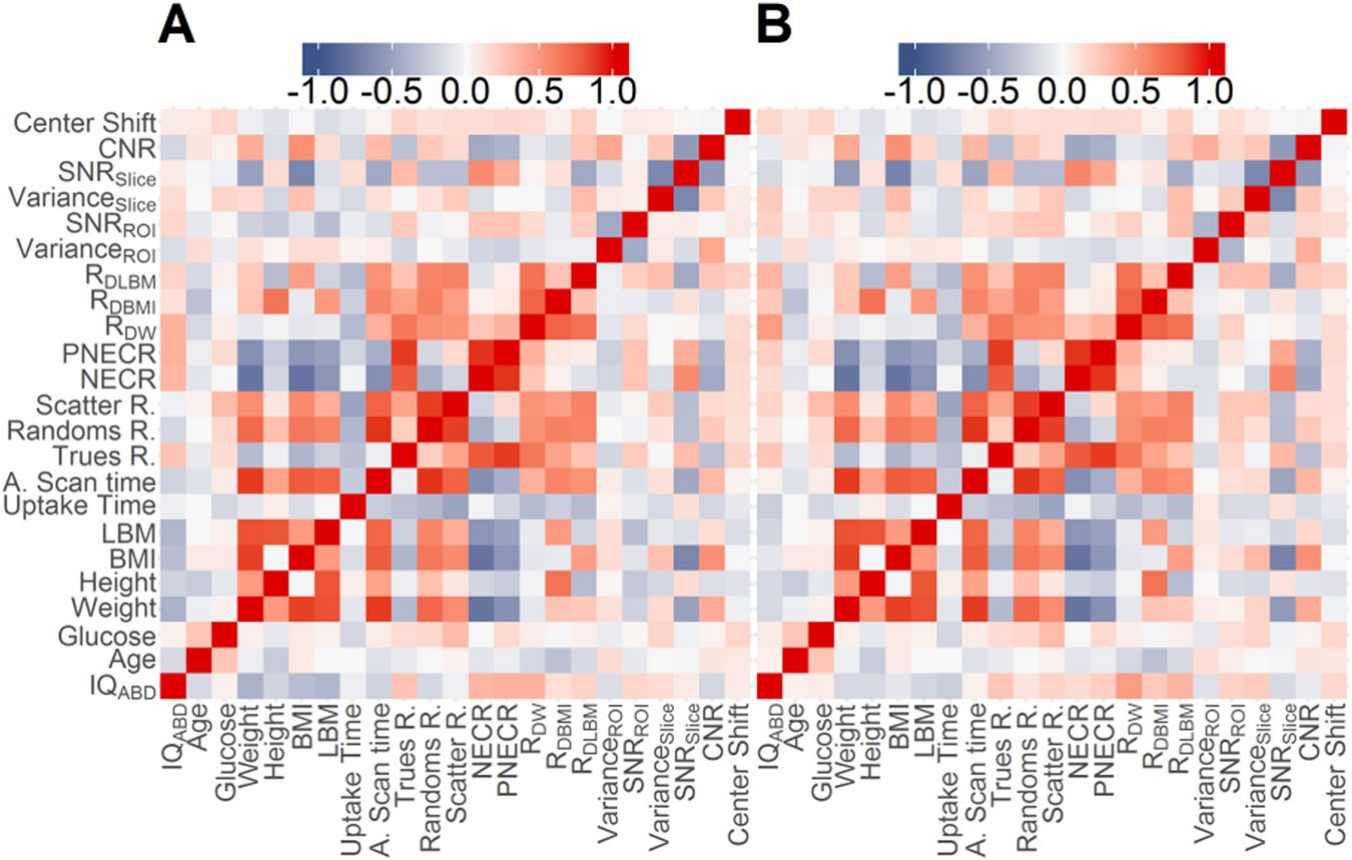
Pearson’s r correlations between all studied variables for OSEM + PSF (A) and BSRM (B) reconstructions. Statistically significant correlations (p < 0.05) are the ones with |r| >0.2.

Mean IQ_ABD_ was 3.0 ± 0.8 and 3.2 ± 0.8 for the OSEM + PSF and BSRM reconstructions, respectively (p = 0.006 using a paired t-test). Weighted Cohen’s kappa coefficient between rankers was 0.46. Figure 2 presents the correlation matrix between all studied variables. For the OSEM + PSF the three IQ parameters presented only at best moderated correlations. The highest correlation coefficients were found with patient weight (r = −0.574), LBM (r = −0.48), BMI (r = −0.41), activity at scan time (r = −0.37), and NECR (r = 0.37). For the BSRM algorithm, correlations between variables and IQ_ABD_ score were lower: R_DW_ (r = 0.43), weight (r = −0.24), and LBM (r = −0.22). For NECR the correlation was also reduced (r = 0.12). On the other hand, despite some observable degree of heteroscedasticity in the data, there was a clear positive correlation between NECR and SNR^2^ (r = 0.54 and 0.56, for the OSEM + PSF and BSRM algorithms, respectively).

**Figure 3:**
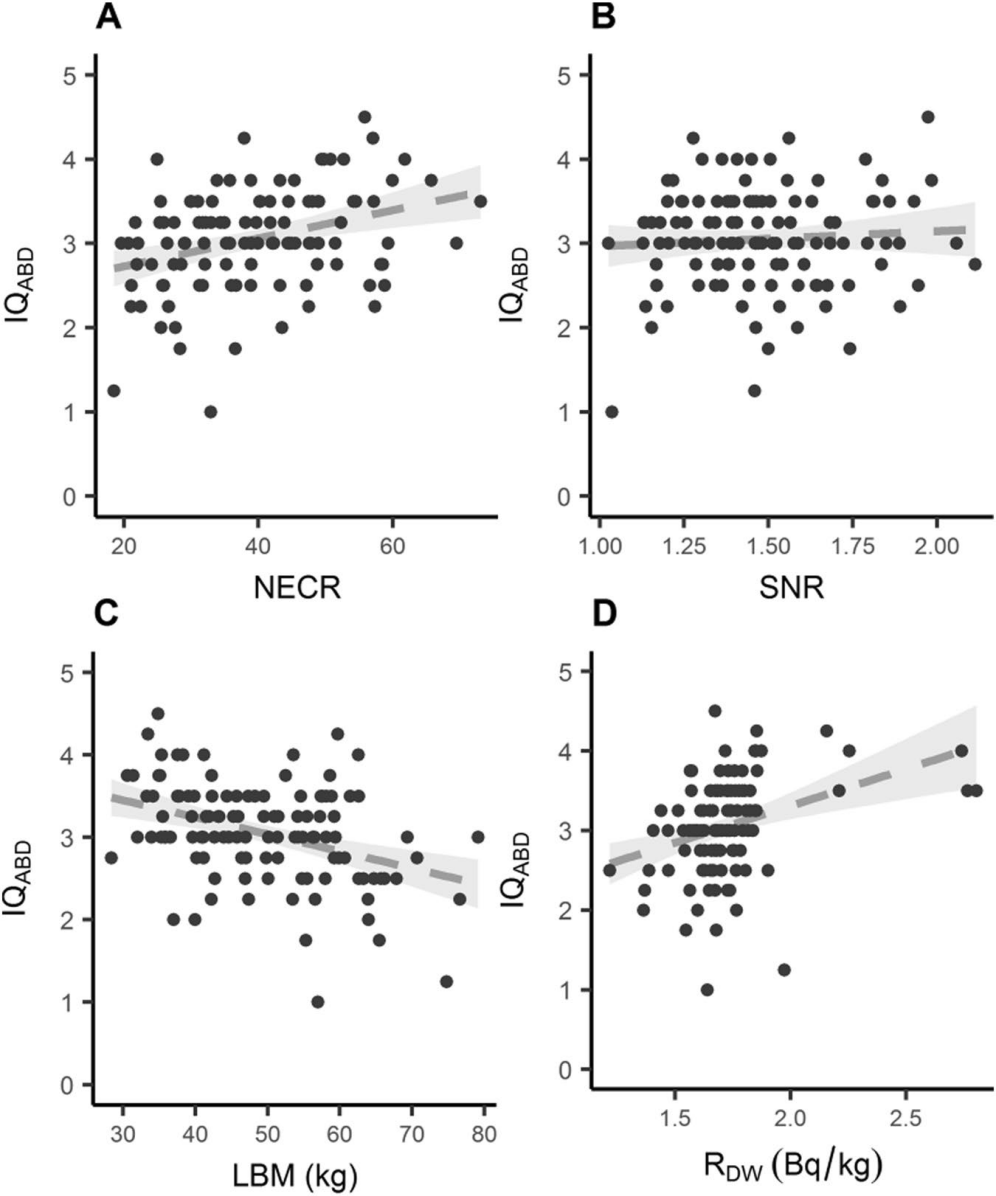
Selected relevant studied variables for the OSEM + PSF (VPDH-S) reconstruction method. The dotted line represents an adjusted linear regression and its 95% confidence interval.

**Figure 4:**
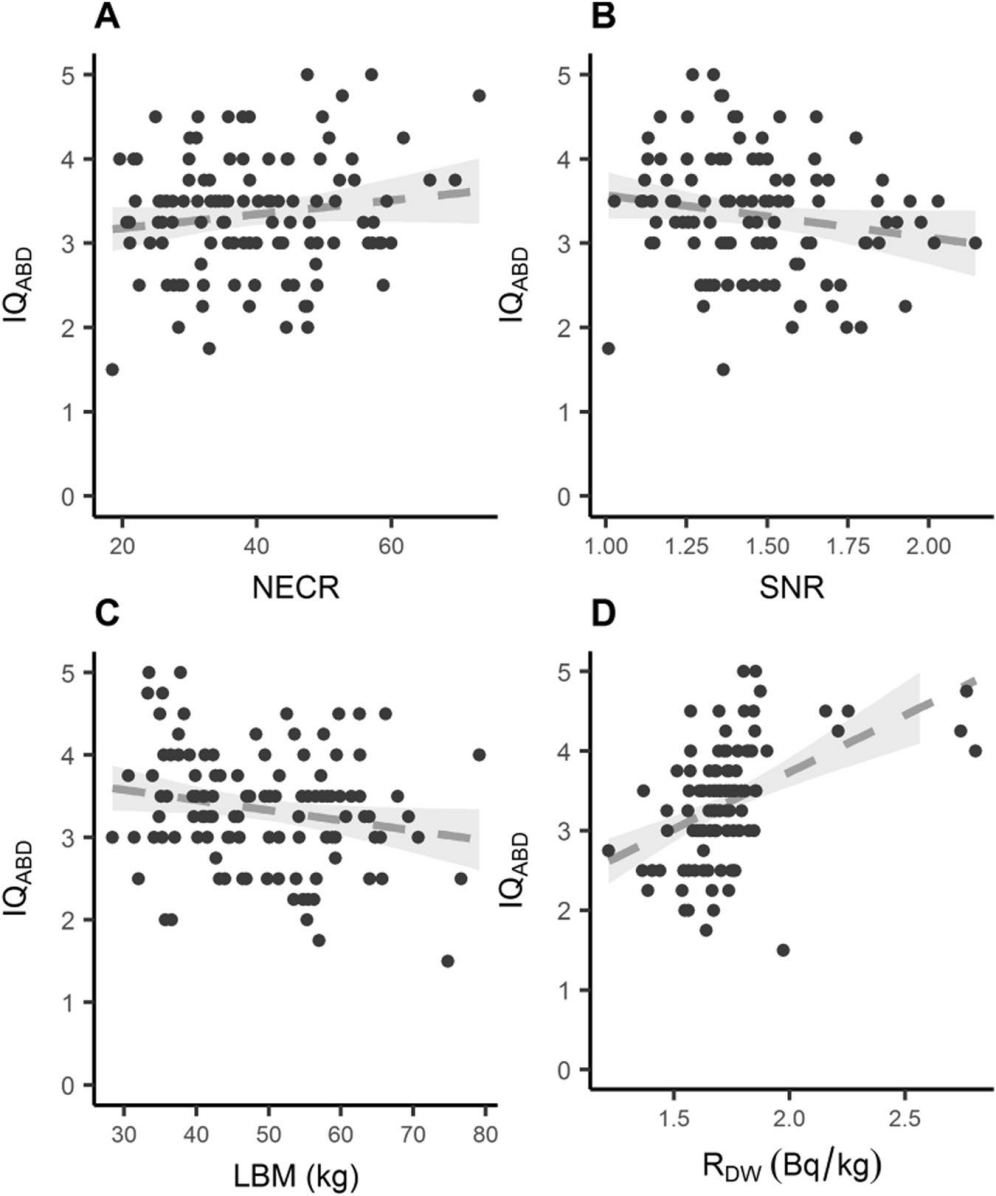
Selected relevant studied variables for the BSRM (Q.Clear) reconstruction method. The dotted line represents an adjusted linear regression and its 95% confidence interval.

Supplemental Fig. 1 shows the relation of NECR with BMI, and Supplemental Fig. 1 the relation of SNR^2^_Slice_ with NECR. It should be noted that R_DW_ presented a highly non-normal distribution of values, and hence the validity of the regression coefficient is limited. Figures 3, 4 present the relation between IQ_ABD_ and some selected variables for both reconstructions.

For the discretized analysis, in the case of the OSEM + PSF reconstruction, the parameters that present statistically significant differences (p < 0.05) are patient LBM (p = 0.0005), R_DW_ (p = 0.007), weight (p = 0.001), height (p = 0.003), CNR (p = 0.01), BMI (p = 0.02), NECR (p = 0.02), and PNECR (p = 0.04). For BSRM the parameters which present lower p-values are patient R_DW_ (p = 0.006), height (p = 0.03), CNR (p = 0.04), and LBM (p = 0.05). For further details, see Supplemental Table 1.

Table 3 shows the AUC obtained from fitting a logistic regression to each statistically significant variable for the train and test dataset. For all parameters, the OSEM + PSF reconstruction presented higher AUC values than the BSRM reconstruction, and from both reconstruction methods, the R_DW_ followed by the LBM parameters presented the highest AUC values for the test data.

**Table 3:** Calculation of the AUC and the 95% confidence interval for all significant variables when using the OSEM + PSF algorithm.

	OSEM + PSF		BSRM	
	Train	Test	Train	Test
Weight (kg)	0.67 (0.50–0.84)	0.64 (0.51–0.78)	0.57 (0.38–0.76)	0.58 (0.44–0.71)
Height (cm)*	0.56 (0.36–0.75)	0.59 (0.44–0.73)	0.51 (0.32–0.70)	0.62 (0.48–0.75)
BMI (kg/m^2^)	0.67 (0.50–0.84)	0.64 (0.49–0.79)	0.56 (0.37–0.75)	0.55 (0.42–0.69)
LBM (kg)*	0.63 (0.44–0.82)	0.65 (0.50–0.80)	0.51 (0.33–0.72)	0.60 (0.47–0.73)
NECR	0.74 (0.57–0.91)	0.65 (0.51–0.78)	0.62 (0.44–0.79)	0.56 (0.42–0.69)
PNECR	0.76 (0.58–0.94)	0.66 (0.53–0.80)	0.60 (0.42–0.78)	0.59 (0.47–0.73)
R_DW_*	0.75 (0.60–0.91)	0.73 (0.61–0.86)	0.75 (0.59–0.91)	0.64 (0.59–0.78)
CNR*	0.54 (0.36–0.73)	0.63 (0.48–0.77)	0.58 (0.39–0.76)	0.53 (0.39–0.66)

Variables that were also significant for the BSRM reconstructions are marked with an asterisk.

**Figure 5:**
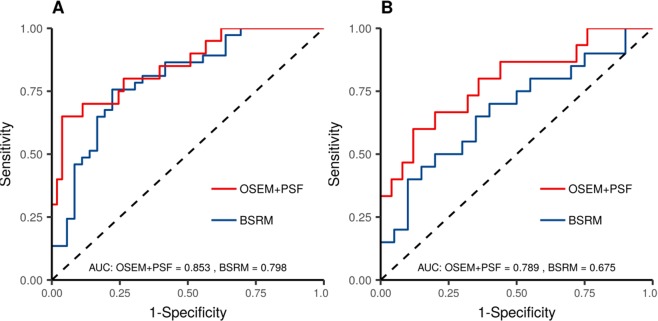
Model performance by means of a ROC curve for (**A**) train and (**B**) test datasets.

Regarding the radiomics model, Supplemental Fig. 1 presents the parameter tuning of the elastic-net model. The resulting ROC can be seen in Fig. 5 for the test and train datasets. The resulting AUC is greater for the OSEM + PSF compared to the BSRM reconstruction. Also, for both algorithms, the radiomics AUC values were higher than the single parameter logistic regressions. The model selected variables and their importance are shown in Supplemental Fig. 4.

## Discussion

The assessment of PET IQ is a complex task, as is highly subjective and depends on many different parameters. This study demonstrates its difficulty when single parameters are used, and aims to point the necessity of adopting an alternative model, as could be a radiomics model, especially when considering the increasing tendency of using penalized algorithms in modern systems. Our work shows how when using modern reconstruction algorithms and clinical acquisition settings most common single parameters are not correlated with the evaluation of IQ by physicians.

Research methodology in the present study is similar to that of Queiroz *et al*.^11^, and in a similar fashion, we found an expected relation between NECR and SNR^2^. Even if our settings were different, especially, to the best of our knowledge, this is the first study evaluating the relationship between NECR and IQ using a BSRM reconstruction algorithm. Despite all this, we did not find any relevant relation between the NECR and IQ scores, particularly for the BSRM reconstruction method. A possible explanation is that our work is restricted to patients with an uptake time below 100 min, instead of the 128.3 min in average in the mentioned paper. Increasing the uptake time could increase the range of NECR values. Our purpose was to use a clinical relevant setting and we restricted our data accordingly. Furthermore, our injected activity is 2.7 MBq/kg instead of 4.3 MBq/kg of Queiroz *et al*.

In accordance to previous publications^15,22^, in our study IQ was also ranked higher for BSRM than for OSEM + PSF. However, when comparing IQ scores with most IQ parameters, lower correlations and higher p-values were found for the BSRM algorithm. This is partially explained by the higher and less variable IQ_ABD_ scores, which limit a possible correlation. Yet, the non-linear reconstruction possibly dismisses the effect of external causes in IQ. The single parameter presenting a higher AUC value for the BSRM algorithm is R_DW_. This result must be taken with caution though, as it presents a non-uniform distribution, as can be seen in the linear regression figures. Thus, the good results in predicting HQ and LQ images could be due a discretization effect, so further work should be performed to confirm its utility. Aside from R_DW_, among all studied parameters, LBM is the only one that shows a lower p-value and a higher AUC in both reconstruction methods.

Most publications about PET IQ using clinical data only focus in lesion conspicuity^3–5^, although there are other independent diagnostic tasks. When dealing with the abdominal zone, parameters such as SNR (extracted from a ROI in the healthy liver) are often used as a measure of IQ^4^, but according to our results, they may have limited value differentiating between LQ and HQ images.

In contrast, we present a simple radiomics model as a proof of concept that a different paradigm can be applied on IQ evaluation, increasing the AUC presented by any single parameter. The present model has several limitations, though. First, this is a retrospective study with a relatively small sample size, even if an independent validation cohort from our institution was used. In the future, a large-scale multicenter study would be convenient to fully assess the generalization ability of the model. Second, it uses extracted data from a single slice and the surrounding slices as different inputs, although the evaluation was performed in the entire abdominal area. Despite the abdominal slice selected was manually verified, in order to increase the number of slices better algorithms for detecting the abdominal area should be applied, as a miss-selection of the abdominal zone could include undesired structures (such as the heart), which could potentially affect any feature values. Third, the model uses an elastic-net algorithm, but other more sophisticated models, such as neural networks could be applied^21^. Moreover, it would be interesting to mix different reconstruction methods and settings in the same model, although that would require a completely different study design, out of the present scope. Lastly, some radiomics features could have a direct interpretation in terms of some IQ traits, such as lesion conspicuity or structure definition. Even if we have treated the model as a black box, it will be still useful to interpret the relation of each radiomics feature with a specific aspect of IQ. It should be noted that to achieve this goal, a current limitation of the present approach is the difficulty to obtain higher correlations between IQ rankers, the present study shows a rather moderate correlation, more work should be done extending redesign the study to include more rankers, ideally from different institutions.

Furthermore, PET IQ is potentially dependent on many pre-imaging parameter conditions^17^, some of which were considered in the present manuscript (i.e. glucose level or uptake-time), but others are difficult to quantify, as could be other metabolic conditions. Additionally, PET imaging has the possibility to modify IQ by changing the acquisition time or reconstruction settings. Beyond the clear advantages of obtaining an objective IQ score, an IQ radiomics model could be performed during the PET scan, by applying a fast OSEM reconstruction during the acquisition, and modifying the duration of the scan or reconstruction settings according to the results. Moreover, our methods are easily extensible to other anatomical areas, such as the brain, where a correct definition of the structures could be of special importance for multicentric clinical trials^20^.

## Conclusion

The present work is a first step to a comprehensive analysis of the abdominal area IQ, pointing the necessity of changing the standard parameters to evaluate IQ, particularly when a BSRM algorithm is involved. Moreover, the promising role of a radiomics approach to assess IQ has been investigated, and according to our results a simple model can outperform the use of any single parameter.

## Supplementary information

Supplemental material
