# Supplementary material for: Image quality evaluation in a modern PET system: impact of new reconstructions methods and a radiomics approach
Source: Sci Rep. 2019 Jul 23;9:10640. doi: 10.1038/s41598-019-46937-8 (PMC6650602; doi:10.1038/s41598-019-46937-8)
Supplement: Supplementary file 1 — Supplemental material [file 41598_2019_46937_MOESM1_ESM.docx]

**Image quality evaluation in a modern PET system: impact of new reconstructions methods and a radiomics approach**

**Gabriel Reynés-Llompart**^1,2^, **Aida Sabaté-Llobera**^2^, **Elena Llinares-Tello**^2^, **Josep M Martí-Climent**^3,*^, **Cristina Gámez-Cenzano**^2^

^1^Medical Physics Department, Institut Català d’Oncologia, L’Hospitalet de Llobregat, Barcelona, Spain

^2^PET Unit. Nuclear Medicine Dept, IDI. Hospital U. de Bellvitge-IDIBELL, L’Hospitalet de Llobregat, Barcelona, Spain

^3^Medical Physics Department, Clínica Universidad de Navarra, Pamplona, Spain

*Corresponding author [jmmartic@unav.es](mailto:jmmartic@unav.es) telf: +34635309523

**Methodology to detect the liver slice**

A heuristic method was used to batch process al PET scans to select the slice comprising a major part of the liver. An initial guess of the slices comprising the live was made (from the second bed to the third). An initial thresholding segmentation was performed to only consider the regions comprising a SUV between 0.1 and 2.6, to avoid lesions or physiologic glucose avid regions (such as heart or bladder).

Next, the slice with the maximum mean SUV was found and the row and columns with the maximum mean SUV where selected as regions of the liver. The correct selection of the liver ROI placement was visually inspected for each patient.

**Additional table and figures**


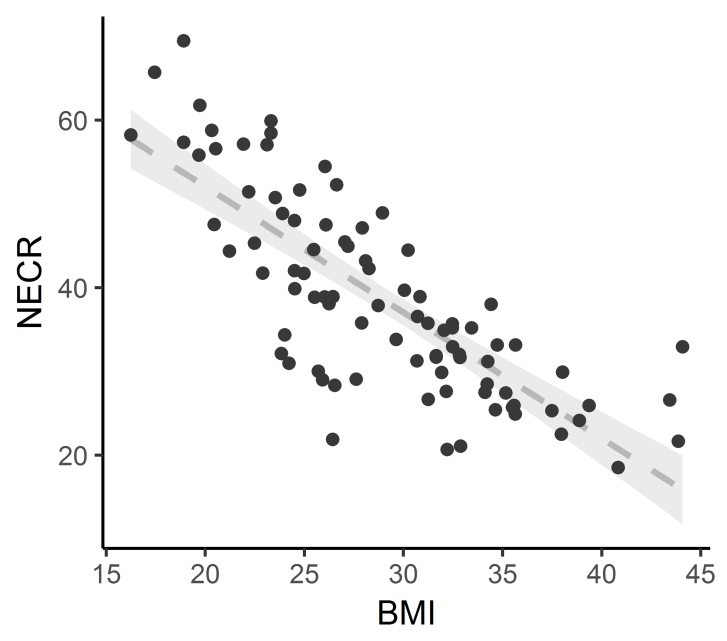


**Supplemental Figure 1.** Relation of NECR and BMI. Dotted line represents and adjusted linear regression and its 95% confidence interval.


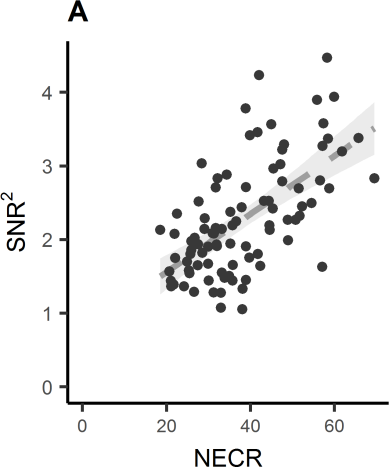

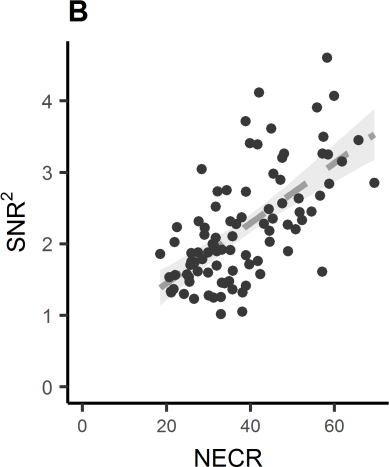


**Supplemental Figure 2.** Relation of NECR and SNR^2^_Slice_ , for a) OSEM + PSF and b) BSRM reconstruction methods. Dotted line represents and adjusted linear regression and its 95% confidence interval.

**Supplemental Table 1.** Studied variables mean and standard deviation for low (LQ) and high (HQ) image quality groups.

|  | OSEM+PSF | | | BSRM | | |
| --- | --- | --- | --- | --- | --- | --- |
|  | LQ | HQ | p-value | LQ | HQ | p-value |
| Patients (n) | 56 | 57 |  | 78 | 35 |  |
| Age (y) | 67±14 | 63±13 | 0.06 | 67±16 | 64±12 | 0.1 |
| Glucose level (mmol/l) | 6±2 | 6±2 | 0.4 | 6.4±1.6 | 6±2 | 0.2 |
| Weight (kg) | 85±21 | 70±14 | 0.001 | 83±20 | 74±17 | 0.1 |
| Height (cm) | 165±8 | 160±10 | 0.003 | 165±8 | 160±10 | 0.03 |
| BMI (kg/m^2) | 31±7 | 27.66±5.77 | 0.02 | 30±6 | 28±6 | 0.17 |
| LBM (kg) | 55±11 | 46±10 | 0.0005 | 53±11 | 48±11 | 0.05 |
| Uptake Time (min) | 71±8 | 71±9 | 0.6 | 72±9 | 70±8 | 0.19 |
| Tracer Activity (MBq) | 140±38 | 123±30 | 0.07 | 136±38 | 127±33 | 0.4 |
| True events | 11±2 | 12±1 | 0.12 | 11E43±20E4 | 12E4±1E4 | 0.17 |
| Random events | 40±15 | 34±12 | 0.11 | 38E4±16E4 | 36E4±1E4 | 0.8 |
| Scatter events | 9±1.8 | 9±1.5 | 0.48 | 9E4±2E4 | 9E4.73±12E4 | 0.9 |
| NECR | 35±12 | 41±12 | 0.02 | 36±11 | 39.63±12.43 | 0.3 |
| PNECR | 111±20 | 121±19 | 0.04 | 112±20 | 118±19 | 0.2 |
| R_DW_ | 1.6±0.1 | 1.7±0.2 | 0.007 | 1.6±0.1 | 1.7±0.2 | 0.006 |
| RD_BMI_ | 4.5±0.6 | 4.5±0.8 | 0.2 | 4.5±0.5 | 4.5±0.8 | 0.7 |
| RD_LBM_ | 2.54±0.4 | 2.7±0.4 | 0.09 | 2.5±0.4 | 2.7±0.5 | 0.3 |
| Variance_ROI_ | 0.08±0.16 | 0.05±0.03 | 0.6 | 0.1±0.3 | 0.05±0.03 | 0.2 |
| SNR_ROI_ | 10±3 | 11±3 | 0.6 | 11±4 | 11±4 | 0.6 |
| Variance_Slice_ | 0.6±0.3 | 0.7±0.2 | 0.3 | 0.7±0.3 | 0.7±0.3 | 0.6 |
| SNR_Slice_ | 1.5±0.2 | 1.5±0.3 | 0.4 | 1.4±0.3 | 1.5±0.3 | 0.6 |
| CNR | 1.6±0.43 | 1.3±0.4 | 0.01 | 1.5±0.4 | 1.3±0.4 | 0.04 |
| Center Shift | 16±10 | 20±12 | 0.2 | 15±9 | 19±11 | 0.7 |

**
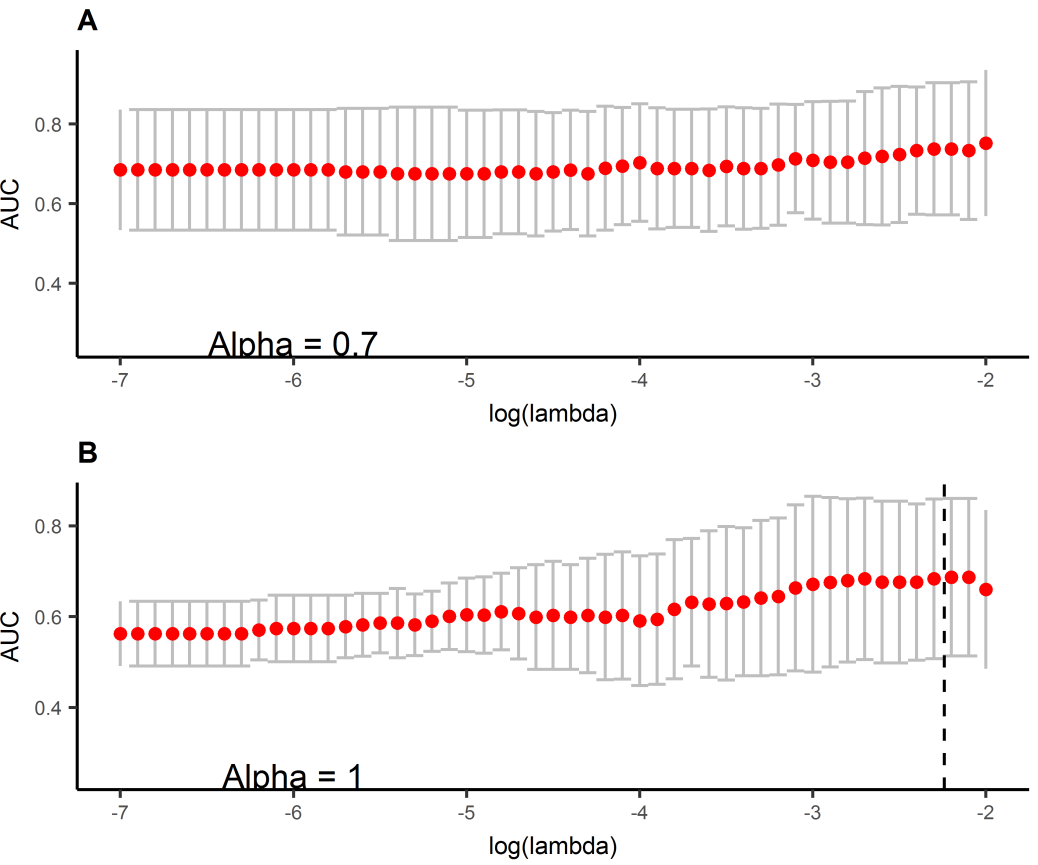
**

**Supplemental Figure 3.** Elastic net lambda optimization by cross-validation.

^
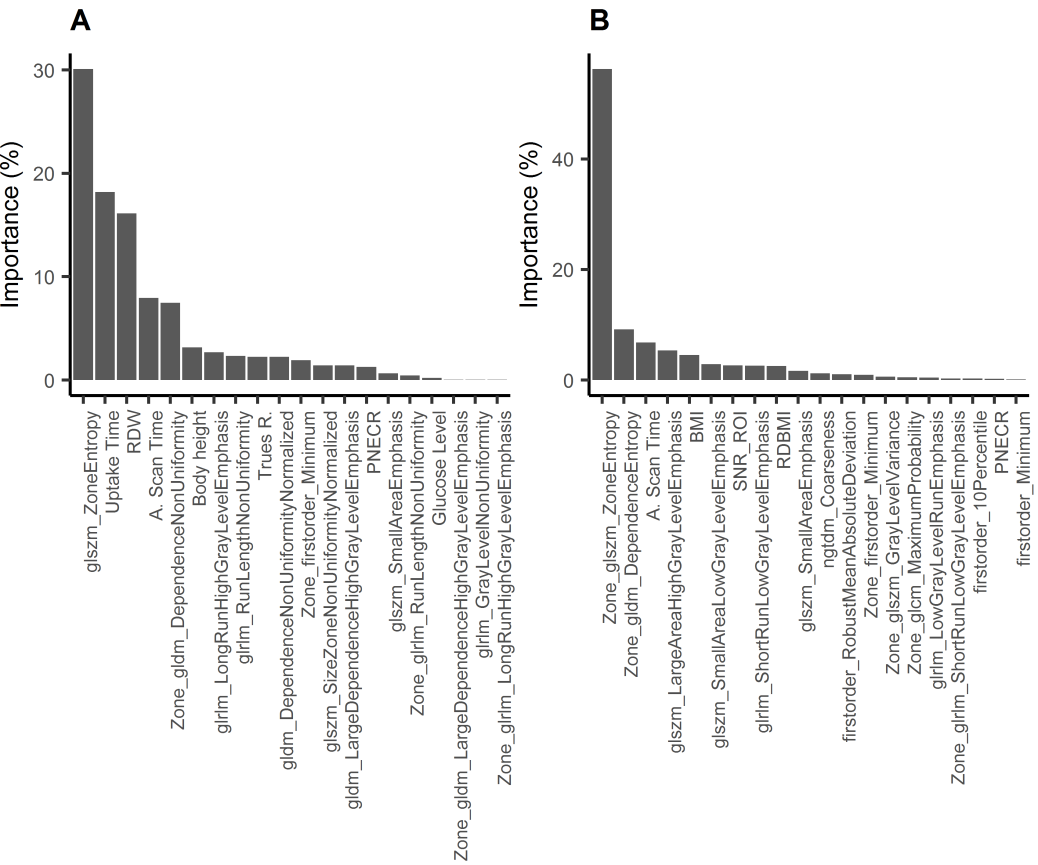
^

**Supplemental Figure 4.** Importance of the selected features for A) OSEM+PSF and B) Q.Clear.
